# Stone-assisted drumming in Western chimpanzees and its implications for communication and cultural transmission

**DOI:** 10.1098/rsbl.2025.0053

**Published:** 2025-05-07

**Authors:** Sem van Loon, Ignas M. A. Heitkönig, Annemarie Goedmakers, Roger Mundry, Marc Naguib

**Affiliations:** ^1^ Behavioural Ecology Group, Wageningen University, Wageningen, The Netherlands; ^2^ Wildlife Ecology and Conservation Group, Wageningen University, Wageningen, The Netherlands; ^3^ Chimbo Foundation, 8567 LL Oudemirdum, The Netherlands; ^4^ Cognitive Ethology Laboratory, German Primate Centre Leibniz Institute for Primate Research, Gottingen, Niedersachsen, Germany

**Keywords:** drumming, tool use, communication, accumulative stone throwing, chimpanzees, animal culture

## Abstract

Chimpanzees (*Pan troglodytes*) communicate in complex ways, including sounds produced by hand and foot drumming on trees, often combined with loud vocalizations. Recently, a puzzling stone throwing behaviour at trees was observed, resulting in stone piles at tree buttresses. It is a rare case of tool used for communication in animals and suggested to function like buttress drumming in long-distance communication and male displays. We tested this hypothesis by determining the behavioural dynamics in comparison to hand and foot tree buttress drumming in Western chimpanzees in Boé, Guinea Bissau. Using camera traps, we show that in 78% of cases, stones were picked up at trees, not leading to further stone accumulation beyond the already existing stone piles. Stone-assisted and hand and foot drumming occurred separately or were combined in similar behavioural contexts in apparent long-distance communication and highly aroused behavioural contexts. Yet, immediately before stone drumming, chimpanzees swayed less and pant-hooted more while afterwards pant-hooting less compared to the other contexts, suggesting a separate motivation and/or function for stone-assisted drumming. It suggests this unique stone-based activity has its own signal value, separate from hand/foot buttress drumming and, considering the spatial variation, might be culturally transmitted.

## 1. Introduction

Communication between and within animal communities and individuals is an essential part of their functioning and survival [1]. Within animal communication, next to vocalizations, vibration and other sounds are widely used and produced by drumming body parts on an object or surface [2,3]. For example, wolf spiders (Lycosidae) drum for sexual display [4,5], tammar wallabies (*Macropus eugenii*), rabbits (*Oryctolagus cuniculus*) and elephant shrews (Macroscelididae) stamp their feet to alarm others [6,7]. Among primates, gorillas (*Gorilla gorilla*) show chest-beating and handclapping [8,9], Tibetan macaques (*Macaca thibetana*) shake branches or knock on dead tree logs [10,11] and humans (*Homo sapiens*) hit walls or slam doors while angry [12]. These primate drumming behaviours are frequently combined with vocalizations, and often seem to be connected to dominance and intimidation [13,14]. The fact that drumming sounds produced on surfaces with low resonant characteristics are usually lower in frequency than vocalizations makes them a prime long-range communication technique as lower frequencies travel over longer distances [15,16]. Chimpanzees (*Pan troglodytes*) produce acoustic drumming signals by hitting tree trunks and buttresses with their hands and/or feet [17,18], often combined with long-distant vocalizations (pant hoots including climax screams), as documented for all studied chimpanzee populations across Africa [19]. Hand and feet drumming is audible over long distances [20] and often combined with vocalizations and male charging displays [21,22]. Several studies thus have concluded that hand and foot tree drumming is used for group spacing and movements, thus also functioning as a long-distance signal [20,22–24].

Recently stone throwing behaviour at trees was described by Kühl *et al*. [25] in some Western chimpanzee (*Pan troglodytes verus*) populations. Rocks are thrown or banged against hollow trees or buttresses, resulting in stone accumulations at the bottom of these trees, termed accumulative stone throwing. Chimpanzees are known to use stones as tools and throw these in various contexts at targets or in water [21,22,26,27], but the accumulative stone throwing at trees appears as a unique characteristic. This behaviour has been observed in only four research sites across the entire range of Western chimpanzees, and it has been suggested to have originated from hand and foot tree drumming behaviour, by incorporating stone tools and evolved into a cultural tradition by social learning [25]. Such tool use in animals is most commonly described in foraging contexts [28]. The behaviour here appears to be used specifically for communication purposes by not only modifying an existing signal [29] but by producing a signal on its own. Kalan *et al*. [30] indeed found that chimpanzees performed stone throwing at tree trunks that are resonating in lower sound frequencies and, therefore, produced louder lower frequency sounds, suggesting that such stone throwing at trees has a similar function as hand and foot tree drumming in long-distance communication.

Yet, to obtain a better understanding of the potential function of stone throwing at trees and its similarity to hand and foot drumming on tree buttresses, along with its potential as a culturally evolved signal, the behavioural and contextual details are needed. If stone throwing at trees is an alternative form of hand and foot tree drumming, we expected to find a long-range communication and/or male-display context. We then expected this to be reflected in associations of stone throwing at trees with long-distance signalling behaviours (pant hoots and climax screams) in highly aroused states and overall low differences in associated behaviours between drumming and stone throwing. If stone throwing at trees emerges out of a different motivation or has a different function, in contrast, we expected behavioural differences preceeding and following stone throwing compared to hand and foot tree drumming. If a persisting main aim is the local accumulation of stones, then we expected chimpanzees to bring new stones, rather than use stones already present at the tree from previous events. Using camera traps, we recorded stone throwing/banging at trees and foot or hand drumming at trees with stone piles, in the Boé sector of Guinea Bissau. We compared the associations of long-distance signalling and male-display behaviours between drumming and stone-assisted drumming in a population of chimpanzees in Guinea Bissau, West Africa.

## 2. Methods

### (a) Study site and data collection

We conducted this study from September to December 2019 in Boé, a forest and savannah area of 3000 km² in south-east Guinea Bissau, where the chimpanzees are not habituated and live in generally undisturbed forests. We included in the analysis camera trap data from additional years (2016−2020 (see electronic supplementary material)). One site had cameras already since 2016 (Tonege), one site since 2017 (Lugajole) and the third site since 2018 (Patte Patte). Chimpanzees have been described anecdotally to show stone-assisted drumming behaviour in various contexts, yet this behaviour has not been analysed with respect to context and the behavioural dynamics [31]. In order to test the actual dynamics and context of stone-assisted drumming, we here selected five drumming tree locations using two criteria: (i) (accumulated) stones lying next to tree trunks or buttresses and (ii) fresh scars and/or tree wound tissue are present on the bark of the buttresses or tree trunk [31]. The locations were Petum Beli, Bundu Njuri Buba, Hore Patte Patte, Hore Lugajole and Tontege (see electronic supplementary material for location details), and each site is characterized by dense vegetation as well as hundreds of stones at each of the observed trees (electronic supplementary material). Around each of the five selected drumming trees, three Bushnell camera traps were placed. The cameras were motion-triggered and then recorded for 60 s unless they were triggered again (15 s at night). Three of the five locations already had one camera since 2017/2018.

### (b) Data analysis

For the behavioural analysis, we used Observer XT software (v. 14.2; Noldus Software, Wageningen, The Netherlands). From the video footage, we first determined whether the performed activity was stone throwing, drumming or the combination thereof, as well as location, individual, time and date. Additionally, we used the videos to quantify the duration of the following behaviours before and after the drumming and/or accumulative stone throwing: (A) far-distant-communication behaviours and contexts: (i) *pant hooting*, (ii) *climax scream*, (iii) *sound surround (vocalizations audible from other chimpanzees)*, (iv) *alert focus environment (apparent active listening)*; (B) stone-related behaviours: (v) *search stone*, (vi) *hold stone*, (vii) *throw or bang stone*; (C) arousal behaviours: (viii) *high activity intensity*, (ix) *piloerection*, (x) *swaying (repeated side-way body movements)*, (xi) *male display rest behaviours (erected penis, leaf clipping, branch shaking, hunched shoulders)*; and (D) locomotion: (xii) *running*, (xiii) *sitting*, and (xiv) *walking*. The focal individual was observed as long as it was visible in the video (max. 60 s) to identify individuals and behaviours. We also coded the use of different trees by different individuals and the intervals between the drumming and stone throwing/banging events.

To estimate the extent to which the scored behaviours varied with respect to the type of activity (i.e. stone throwing, drumming, or the combination of the two), we fitted a series of five generalized linear mixed models [32], respectively, before and after the drumming/stone event. Since we had very few observations of females and non-adult individuals, we only included observations of adult males with known identify and complete information. For these we compared whether the individuals pant-hooted (model 1), climax screamed (only after, see below, model 2), were piloerected (model 3), swayed (only before, see below, model 4), other calling chimpanzees were present (sound surround; model 5) and/or showed apparent active listening (alert focus environment, model 6). We fitted these behaviours in two models, one with whether or not the behaviour occurred before the onset of drum/stone event (no or yes) and one with whether or not the behaviour occurred afterwards. Climax screaming was modelled only afterwards (as beforehand it occurred only once) and swaying was modelled only beforehand (as afterwards it occurred only four times). We also repeated these analyses, not separating between occurrence before or occurrence after the signalling behaviour (see electronic supplementary material for details).

We selected these six behaviours because the others occurred very rarely. In all models, the response was whether or not a given behaviour occurred. Hence, we fitted the models with a binomial error distribution [32] and logit link function [33]. The key fixed effects predictor in all models was the type of tree-directed activity (stone throwing, drumming or the combination of both), and as a grouping factor with random intercepts effect, we included the identity of the observed individual. As the far majority of the individuals were observed with a single tree-directed activity only, a random slope [34] of activity type within the individual was not identifiable.

To account for the varying durations of the time the individuals were visible (range: 1 s to 1 min and 1 s), we included the observation duration as an offset term. To this end, we used a self-written function, which sets the offset term as O = 1 – (1 – *B*^observation duration) and included it into the model formula as offset(log(O / (1 – O))). The function estimates *B* such that the log-likelihood of the observed response, given the predictors and the fitted model (including the fixed and random effect), is maximized.

We fitted the models in R (v. 4.3.2 [35]) using the function glmer of the package lme4 (v. 1.1.35 or higher [36,37]). The optimization of the value of *B* was done with the aid of the function optimization. We determined the significance of signal type by dropping it from the model and comparing the reduced model with the respective full model using a likelihood ratio test [38]. To estimate the confidence limits of model estimates and fitted values, we used a parametric bootstrap (*n* = 1000 bootstraps; function bootMer of the package lme4). We determined model stability by dropping individuals from the data, one at a time, fitting the model to each of the respective subsets and comparing the estimates derived with those obtained for the full data set. This revealed all models to be of moderate to good stability (see results). We kept the value of *B* constant during the stability assessment. The samples analysed with these models comprised a total of 135 signal contexts observed for 28 individuals (before models) and 155 signal contexts observed for 27 individuals (after models).

With an additional model we investigated the extent to which chimpanzees exhibited preferences for certain signal types at certain sites. To this end we fitted a multinomial mixed model in the Bayesian framework made available with the R package brms (v. 2.21.0 [39,40]; for more details see the electronic supplementary material).

## 3. Results

Before the onset of stone throwing and/or drumming, the chimpanzees usually displayed low-intensity activities. In the case of stone throwing, chimpanzees often searched and handled a stone, but in 78.3% of the stone throwing, the stone was not brought from elsewhere. Then, both during stone throwing and drumming, the activity intensity increased, combined with preceding pant hoots until the stone was thrown or banged against the tree and/or hands and feet were drummed against the tree. During and just after a throw/drum bout, climax screams in high intensity were frequently observed. Afterwards, the chimpanzees swayed more but overall became less active, often appeared to be listening, and far-distant chimpanzees were often heard vocalizing. Commonly, piloerection was visible throughout the whole behavioural sequence.

**Figure 1.**
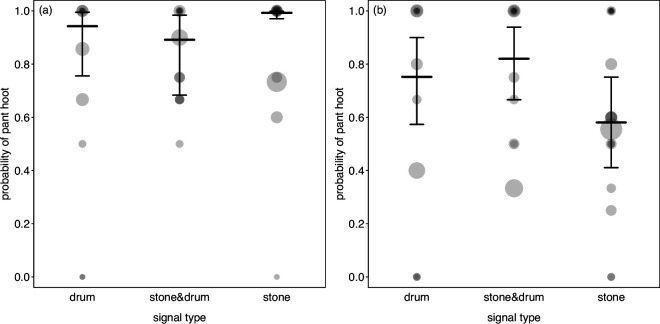
Likelihood of occurrence of pant hoots accompanying just drumming, the combination of drumming and stone throwing, or just stone throwing, beforehand (a) and afterwards (b). Dots show the empirical probability per individual and behavior type whereby the area of the dots is proportionate to the number of events per individual and behavior type (range: 1 to 18) and darker dots indicate overlapping data points. Horizontal line segments with error bars depict the fitted model and its 95% confidence intervals.

**Figure 2.**
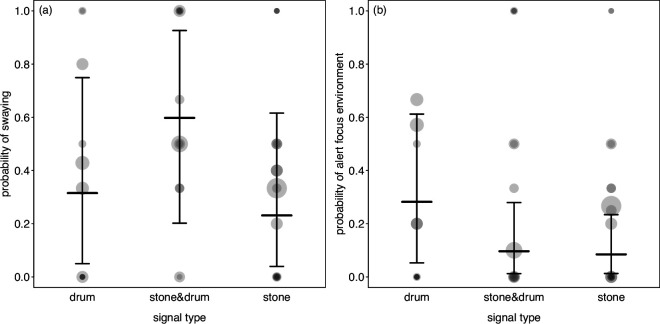
Likelihood of the occurrence of swaying (a) and alert focus environment (b) accompanying just drumming, the combination of drumming and stone throwing, or just stone throwing before the event. Dots show the empirical probability per individual and signal type whereby the area of the dots is proportionate to the number of signalling events per individual and signal type (range: 1 to 15), and darker dots indicate overlapping data points. Horizontal line segments with error bars depict the fitted model and its 95% confidence intervals.

**Figure 3.**
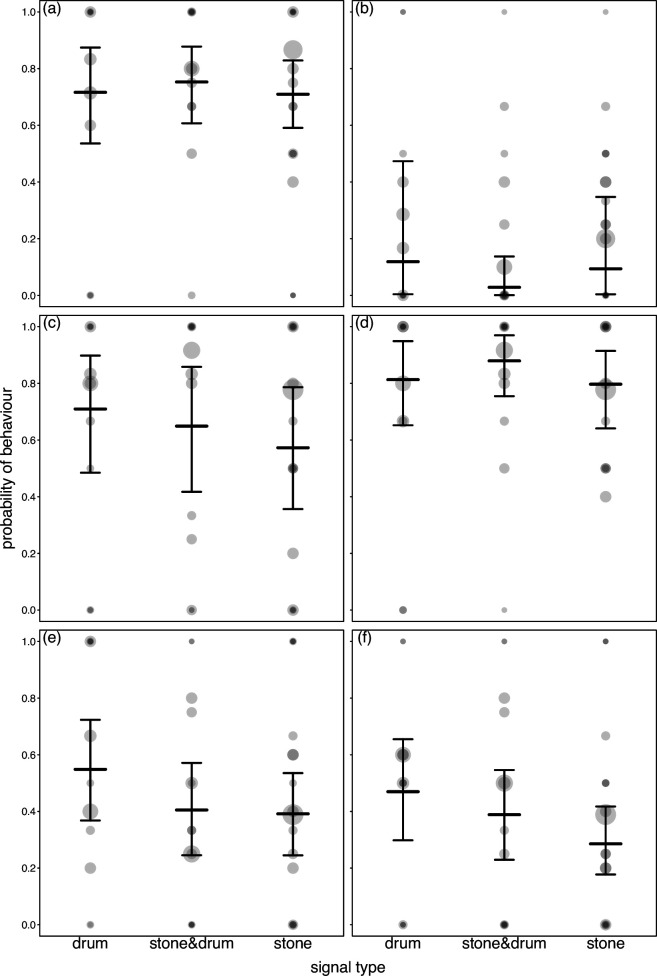
Likelihood of occurrence of piloerection before the event (a), sound surround beforehand (b), climax screams afterwards (c), piloerection afterwards (d), sound surround afterwards (e) and alert focus environment afterwards (f), accompanying just drumming, the combination of drumming and stone throwing, or just stone throwing. Dots show the empirical probability per individual and signal type whereby the area of the dots is proportionate to the number of events per individual and signal type (range: 1 to 18), and darker dots indicate overlapping data points. Horizontal line segments with error bars depict the fitted model and its 95% confidence intervals.

Two of the 10 statistical models testing for the different behaviours, revealed a significant effect of the activity type (stone throwing, drumming or the combination), and in two more, we found a marginally non-significant effect of the activity type (table 1). More specifically, before the event, pant hoots were more likely when stone throwing followed as compared to drumming or the combination of stone throwing and drumming (figure 1a, table 1). Furthermore, afterwards, pant hoots were less common after stone throwing compared to drumming or the combination of stone throwing and drumming (figure 1b, table 1). In addition, beforehand swaying tended to be more common when the combination of stone throwing and drumming followed (figure 2a, table 1), and an apparent alert focus on the environment tended to be more likely when drumming followed (figure 2b, table 1). In none of the other models, we found a significant effect of drumming or stone use. Plotting the respective data and model results revealed that in all these combinations of behaviour and period (before or after drum/stone) the variation between individuals observed with the same category (drum/stone/combination) was much larger than the variation between them (figure 3). When repeating these analyses without separating between whether behaviours occurred before or after the signalling event, we found no evidence for the probability of occurrence of the different behaviours to differ between signals comprising only drums, only stone throwing, or the combination of them (see electronic supplementary material for details).

**Table 1.** Results of all binary models of whether or not a given behaviour occurred, before and after signalling. Indicated are estimates, together with their standard errors, 95% confidence limits, significance tests and the range of estimates obtained when dropping individuals one at a time. Activity type was dummy coded with drumming being the reference level. The estimates for Beh. St & Dr and Beh. St indicate the estimated magnitude and direction of the difference between the probabilities of the respective behaviour to occur when chimpanzees drummed and threw stones (Beh. St & Dr) or just threw stones (Beh. St) respectively, on the one hand, and just drummed, on the other hand. The indicated significance tests refer to the overall effect of behaviour type. Each model included a random intercepts effect for the ID of the individual, and the response variable in a given model was whether the behaviour (indicated in the column ‘response’) occurred (no or yes) before or after the signalling event (column ‘when’).

when	response	term	Est.	SE	Cl_lower_	Cl_upper_	χ2	d.f.	P	min	max
before	pant hoot	intercept	5.919	0.988	4.251	8.447				4.215	6.874
		Beh. St & Dr	−0.690	1.043	−3.062	1.499	9.743	2	0.008	−1.675	1.096
		Beh. St	2.105	1.068	−0.442	4.131				1.079	5.212
	piloerection	intercept	−0.144	0.391	−0.927	0.870				−0.358	0.114
		Beh. St & Dr	0.188	0.533	−1.000	1.300	0.245	2	0.885	−0.070	0.488
		Beh. St	−0.033	0.485	−1.185	0.938				−0.316	0.158
	swaying	intercept	2.197	0.914	0.026	4.067				1.529	2.631
		Beh. St & Dr	1.172	0.779	−0.621	3.203	5.466	2	0.065	0.813	2.463
		Beh. St	−0.425	0.860	−2.449	1.498				−1.084	0.361
	sound.surround	intercept	0.929	0.907	−2.616	2.827				0.417	1.392
		Beh. St & Dr	−1.536	0.880	−3.870	0.504	3.975	2	0.137	−2.416	−1.047
		Beh. St	−0.265	0.911	−2.298	1.929				−0.842	0.870
	alert.focus.environment	intercept	−3.512	0.675	−5.465	−2.121				−4.354	−3.279
		Beh. St & Dr	−1.303	0.711	−2.998	0.136	4.906	2	0.086	−1.751	−0.246
		Beh. St	−1.444	0.730	−3.145	0.082				−1.827	−0.224
after	pant hoot	intercept	0.043	0.479	−0.770	1.127				−0.253	0.315
		Beh. St & Dr	0.410	0.528	−0.799	1.684	6.134	2	0.047	0.181	0.797
		Beh. St	−0.784	0.514	−1.982	0.112				−1.029	−0.487
	climax.scream	intercept	1.070	0.568	0.115	2.351				0.872	1.289
		Beh. St & Dr	−0.278	0.600	−1.443	0.844	1.091	2	0.580	−0.708	−0.012
		Beh. St	−0.600	0.578	−1.808	0.505				−0.862	−0.378
	piloerection	intercept	0.405	0.542	−0.437	1.858				0.159	0.703
		Beh. St & Dr	0.516	0.608	−0.796	1.973	1.316	2	0.518	0.212	0.788
		Beh. St	−0.102	0.555	−1.395	1.049				−0.364	0.127
	sound.surround	intercept	0.335	0.377	−0.401	1.101				0.069	0.589
		Beh. St & Dr	−0.579	0.464	−1.542	0.295	2.281	2	0.320	−0.894	−0.354
		Beh. St	−0.636	0.447	−1.601	0.230				−0.945	−0.436
	alert.focus.environment	intercept	0.121	0.378	−0.614	0.883				−0.013	0.370
		Beh. St & Dr	−0.333	0.450	−1.310	0.500	3.207	2	0.201	−0.537	−0.145
		Beh. St	−0.795	0.442	−1.723	0.061				−0.992	−0.662

For all three contexts (stone/drum/combination), identified individuals (*n* = 16) returned to the same tree for subsequent stone throwing and/or drumming, with intervals varying from seconds (*n* = 15), to minutes (*n* = 25), hours (*n* = 13), days (*n* = 37) and even months (*n* = 9). The relative frequency with which the accompanying behaviours were associated with drumming, stone throwing and the combination of the two did not differ much between signal types (electronic supplementary material, figure SI 1), Furthermore, the relative frequency of occurrence of stone throwing, drumming and their combination varied in part considerably between locations and individuals (electronic supplementary material, figure SI 2), and for the one individual that was observed at different locations, it also varied between locations.

## 4. Discussion

Here, we show that stone throwing at trees in chimpanzees was performed along with clear long-distance communication and male display signalling behaviours in highly aroused states. We show that hand and foot tree drumming and stone throwing activities had similar behavioural dynamics, were often combined, yet differed with regards to pant hooting and swaying before and after the stone throwing compared to drumming. These findings expand on previous observations [25] suggesting that stone throwing at trees in Western chimpanzees is part of a communication system by now showing that its performance is more than a modified form of drumming, as reflected in different behavioural dynamics.

Generally, stone throwing activities appeared to have similar dynamics as hand and foot tree buttress drumming, both being sometimes combined in the same sequence, and both being characterized by aroused behaviour, such as pant hoots and swaying (also see analysis in the electronic supplementary material). Thus, functionally, both appear to have communicative purposes in long-distance group spacing and/or male display [21,22,41–43]. Yet the fact that chimpanzees pant-hooted more and swayed less before stone throwing, but pant-hooted less afterwards, in comparison to drumming or the combination of the two behaviours, suggests that stone throwing develops out of a different state. Even though less swaying before a stone throw could be explained by the fact that it will be more difficult to sway while carrying a stone, the differences in pant hooting suggests that stone throwing may have a different function than drumming within the wider similar context, possibly enhancing long-range communication effects rather than local intimidation.

Moreover, our findings, along with those of Babiszewska *et al*. [41] and Arcadi *et al*. [20], suggest that different drumming patterns together with pant hoots, may act as individually distinctive long-distance signals that could provide information about individual identity and/or the context. Since primarily adult males performed stone throwing, which was often combined with additional male display behaviours, we suggest that the physical ability of adult males to give a heavy stone the speed required to produce a loud noise could indicate strength, which therefore, could enhance the effectiveness of male display. Additionally, a benefit of introducing stones in drumming could be that throwing or banging a heavy stone against a resonating tree, instead of only hand and feed drumming, could have sound transmission advantages [16]. Indeed, Kalan *et al*. [30] tested impact sounds produced by throwing rocks at trees and showed that the buttresses of trees used for stone throwing produce low-frequency sounds which travel further in the environment.

Kühl *et al*. [25] termed the behaviour ‘accumulative stone throwing’ and suggested that it could be functioning to make symbolic rock accumulations or territorial land markings. Even though that might well be the case at an initial stage, our findings show, as well as those of Kühl *et al.*, that at trees with large stone piles, most of the times stones were picked up from the tree base itself and not brought from elsewhere. This suggests that stone accumulation is not a persisting goal once some level of accumulation has taken place. Our findings thus suggest that ‘stone-assisted drumming’ might be a better term reflecting this behaviour than does accumulative stone throwing. Kühl *et al*. [25] further suggested that stone throwing/banging at trees has culturally evolved as a variant of hand and feet drumming behaviour. Various studies indeed showed that variation in tool-use and communication in chimpanzees can be explained by culture and social learning [44–47]. Our findings on the strong variation among locations and individuals in the performance of stone-assisted drums, hand and foot tree drumming, and the combination thereof, indeed are in line with the idea of a culturally transmitted behaviour as this variation is a basic requirement for cultural evolution to act on [44,48,49]. The fact that the locations of stone-assisted drums are very distinct and across a geographical scale where also other tool use can differ [44], supports the idea that the observed spatial differences have a culturally evolved component. Additionally, the current study found performances of non-adult chimpanzees, which are described by Buys *et al*. [31] during which young individuals appeared to copy stone-assisted drumming, which could suggest social learning, another criterion for cultural evolution [31,50–54].

To create a better understanding of the cultural evolution of stone-assisted drumming, further research about spatial, individual and environmental variation of the behaviour would be important. It is also one of the rare cases in which tools are being used for communication purposes rather than for foraging and/or to solve another immediate problem [28]. Stone-assisted drumming, a modified form of hand and feet drumming behaviour, has the potential to become a prime example to understand culturally evolved animal communication behaviours.

## Data Availability

Data available from the Dryad Digital Repository [55]. Supplementary material is available online [56].
